# The Generic Short Patient Experiences Questionnaire (GS-PEQ): identification of core items from a survey in Norway

**DOI:** 10.1186/1472-6963-11-88

**Published:** 2011-04-21

**Authors:** Ingeborg Strømseng Sjetne, Oyvind A Bjertnaes, Rolf Vegar Olsen, Hilde Hestad Iversen, Geir Bukholm

**Affiliations:** 1 Norwegian Knowledge Centre for the Health Services, P.O.box 7004 St Olavs Plass, N-0130 Oslo, Norway; 2 Department of Teacher Education and School Research, University of Oslo, P.O.box 1099 Blindern, N-0317 Oslo, Norway; 3 Institute of Health and Society, University of Oslo, P.O.box 1078 Blindern, N-0316, Oslo, Norway

## Abstract

**Background:**

Questionnaires are commonly used to collect patient, or user, experiences with health care encounters; however, their adaption to specific target groups limits comparison between groups. We present the construction of a generic questionnaire (maximum of ten questions) for user evaluation across a range of health care services.

**Methods:**

Based on previous testing of six group-specific questionnaires, we first constructed a generic questionnaire with 23 items related to user experiences. All questions included a "not applicable" response option, as well as a follow-up question about the item's importance. Nine user groups from one health trust were surveyed. Seven groups received questionnaires by mail and two by personal distribution. Selection of core questions was based on three criteria: applicability (proportion "not applicable"), importance (mean scores on follow-up questions), and comprehensiveness (content coverage, maximum two items per dimension).

**Results:**

1324 questionnaires were returned providing subsample sizes ranging from 52 to 323. Ten questions were excluded because the proportion of "not applicable" responses exceeded 20% in at least one user group. The number of remaining items was reduced to ten by applying the two other criteria. The final short questionnaire included items on outcome (2), clinician services (2), user involvement (2), incorrect treatment (1), information (1), organisation (1), and accessibility (1).

**Conclusion:**

The Generic Short Patient Experiences Questionnaire (GS-PEQ) is a short, generic set of questions on user experiences with specialist health care that covers important topics for a range of groups. It can be used alone or with other instruments in quality assessment or in research. The psychometric properties and the relevance of the GS-PEQ in other health care settings and countries need further evaluation.

## Background

Collection of patient reported outcomes, including patient experiences, is an important component of health services evaluation. According to a recent review [1], several countries have programs for monitoring health care quality using surveys that inquire into patients' and other health care users' experiences. The surveys have different target populations such as the general population [2], users of specific services [3], or patients with specific conditions [4]. The recipients of the results, and hence the use of the results, also vary among the programs between national health authorities, health care managers at different levels, health insurers, providers, potential service users, and researchers. Depending on the survey design, the results can be used to monitor health system performance and/or inform quality improvement efforts at the level of service delivery.

As in many of the reviewed surveys [1], data are collected using lengthy, printed questionnaires in Norwegian national patient experiences surveys. Questions have been raised regarding the burden in collecting (both for respondents and collectors), analysing, and reporting the data versus the usefulness of the survey results [5]. Therefore, many instruments have been modified to short forms to reduce this burden, and to make it possible to use more than one instrument in survey questionnaires (e.g., tools to measure generic and condition-specific health-related quality of life [6,7] and patient experiences in general practice and in hospitals [8,9]). However, the methods used for shortening questionnaires vary. The Medical Outcomes Study Short Form (SF-36) was shortened to the SF-8 based on a combination of content coverage issues and an empirical assessment of the correlation between the short and long form [7]. The original instrument developed by the European Task Force on Patient Evaluations of General Practice (EUROPEP) was reduced to the EUROPEP 2006-SF10 version based on factor analysis and an assessment of item discrimination [8]. Factor analysis was used in the development of instruments such as the WHO responsiveness instrument [10] and the Quality from the Patient's Perspective questionnaire (QPP) [9]. The respondents' evaluation of importance was also considered when deciding which items to retain in the QPP.

In 2008, the Norwegian Knowledge Centre for the Health Services (NOKC) received a request from a specialist health care trust about constructing a generic short set of items to be based on the NOKC's previous work. The trust's objective was to use the constructed set of items in a practical, local routine for the continuous collection of patients' and other users' reports on quality. A generic tool would make it possible to approach different target populations with the same items, and a brief format would ease the response burden and was expected to facilitate a good response rate [11]. Limiting the number of items to a maximum of ten would allow testing other questionnaire formats in addition to the traditional printed booklet, such as a single sheet of paper or touch-screens.

The NOKC has developed and assessed a family of questionnaires for six target populations in different specialist health care services [12-18]. These instruments have been used in a series of national surveys of patient experiences in Norway, and were the starting point for the present project.

As there are no standardised methods for shortening questionnaires, we built upon international literature to develop a set of criteria to identify core items including relevance, importance, and comprehensiveness. We conducted a survey among nine user groups who had had recent contact with the health trust, asking a number of user experiences questions and also asking, for each question, about the importance of the question's content.

The purpose of this paper is to describe the selection of ten generic core questions that cover the essential dimensions of users' experiences with the services provided across a range of specialist health care services. Question scores are intended to be used as single indicators for each of the ten specific content areas.

## Methods

### Setting

All data were collected in 2008 from patients admitted to Helse Bergen health trust. The trust included a university hospital that delivered local and regional specialist health care in the somatic and psychiatric sector and employed 7600 full-time equivalents. Alcohol and substance dependency care was delivered by agreement between the health trust and a private foundation.

### Sample selection

Nine groups of care users were included and constituted subsamples: 1) outpatients undergoing rehabilitation; 2) out- and 3) inpatients in somatic care; 4) out- and 5) inpatients in psychiatric care; 6) out- and 7) inpatients in alcohol and substance dependency care, 8) next-of-kin to children in inpatient somatic care; and finally, 9) next-of-kin to children in outpatient mental care. Analyses performed by NOKC on data from previous similar surveys showed that a final sample of 200 to 400 respondents in each group would be good, and 50 to 100 would be acceptable [19]. Therefore, the objective was to obtain 200 respondents from each group.

Staff at the institutions delivered the questionnaires on site to the patients in alcohol and substance dependency care. The trust sent all the other patients and service users the questionnaire by mail at their home address.

### Original questionnaires

Candidates for core items were chosen from the established family of instruments (see Table 1). The development and assessment of the six original questionnaires is documented in detail separately. The Patient Experience Questionnaire (PEQ) for adult, somatic inpatients was the first questionnaire to be developed [12] and PEQ was the basis for the later development of the Nordic Patient Experiences Questionnaire (NORPEQ) for inpatients (aged ≥ 16 years) discharged from somatic care [13]. The Outpatient Experiences Questionnaire (OPEQ) was developed for outpatients (aged ≥ 16 years) in somatic clinics and day units [14], the Psychiatric Inpatient Experiences Questionnaire (PIPEQ) for psychiatric inpatients (aged ≥ 18 years) [15], the Psychiatric Outpatient Experiences Questionnaire (POPEQ) for psychiatric outpatients (aged ≥ 18 years) [16], the Parents Experiences of Paediatric Care (PEPC) for reporting the experiences of next-of-kin of children under 16 years in paediatric inpatient care [17], and the Parent Assessment of Outpatient Child and Adolescent Mental Health Services (CAMHS) for reporting the experiences of next-of-kin of children under 16 years in outpatient psychiatric care [18].

**Table 1:** Origins of the generic questions (dimension in the source questionnaires) and their status in the ten-item version

	Generic questions	Source questionnaire (Original dimension)	Selected question
4	Did the *clinicians *talk to you in a way that was easy to understand?	• NORPEQ^a ^(Unidimensional) • OPEQ^b ^(Communication) • PEPC^c ^(Physicians) • CAMHS^d ^(Clinicians)	X

5	Do you have confidence in the *clinicians' *professional competence?	• NORPEQ^a ^(Unidimensional) • OPEQ^b ^(Communication) • PEPC^c ^(Physicians)	X

6	To what degree did you perceive that the *clinicians *cared about you?	• NORPEQ^a ^(Unidimensional) • OPEQ^b ^(Communication) • PEPC^c ^(Physicians) • CAMHS^d ^(Clinicians)	

7	Did you perceive the *clinicians *to be interested in your description of your situation?	• NORPEQ^a ^(Unidimensional) • PIPEQ^e ^(Unidimensional) • OPEQ^b ^(Communication) • POPEQ^f ^(Clinician interaction) • PEPC^c ^(Physicians) • CAMHS^d ^(Clinicians)	

8	Did you get enough time to talk and interact with the *clinicians?*	• PIPEQ^e ^(Unidimensional) • OPEQ^b ^(Communication) • POPEQ^f ^(Clinician interaction) • CAMHS^d ^(Clinicians)	

9	Did the *other staff *talk to you in a way that was easy to understand?	• PEPC^c ^(Nurses)	


10	Do you have confidence in the *other staff's *professional skills?	• NORPEQ^a ^(Unidimensional)	

11	To what degree did you perceive that the *other staff *cared about you?	• NORPEQ^a ^(Unidimensional) • PEPC^c ^(Nurses)	

12	Did you perceive the *other staff *to be interested in your description of your situation?	• NORPEQ^a ^(Unidimensional) • PIPEQ^e ^(Unidimensional) • PEPC^c ^(Nurses)	

13	Did you get enough time to talk and interact with the *other staff*?	• PIPEQ (Unidimensional)	

14	Were you told as much as you considered necessary about how tests or examinations would be carried out?	• NORPEQ^a ^(Unidimensional) • OPEQ^b ^(Information) • PEPC^c ^(Information - tests)	

15	Did you get sufficient information about your diagnosis/your afflictions?	• PIPEQ^e ^(Unidimensional) • OPEQ^b ^(Information) • POPEQ^f ^(Information) • PEPC^c ^(Information - tests)	X

16	Did you perceive the treatment you received as suited to your situation?	• PIPEQ^e ^(Unidimensional) • POPEQ^f ^(Clinician interaction)	X

17	Were you involved in any decisions regarding your treatment?	• PIPEQ^e ^(Unidimensional) • OPEQ^b ^(Information) • POPEQ^f ^(Clinician interaction) • CAMHS^d ^(Information and involvement)	X

18	Did you perceive the institution's work as well organised?	• OPEQ^b ^(Organisation) • PEPC^c ^(Organisation)	X

19	Did you perceive that the institution prepared you for the time after the treatment was finished?	• PIPEQ^e ^(Unidimensional) • OPEQ^b ^(Information) • PEPC^c ^(Information - discharge)	

20	Do you find that the institution has co-operated well with other public services (e.g. your GP, NAV^f^, or district nurse)?	• An extension of item 19 to capture also activities that facilitate service co-ordination across levels of care	

21	Did you have to wait before you were admitted for services at the institution?	• OPEQ^b^, POPEQ^f^, CAMHS^d^. Single item increasingly used for comparison with administrative data	X

22	Did you get the impression that the hospital equipment was in good order?	• OPEQ^b ^(Standard) • PEPC^c ^(Standard)	

23	Did you get the impression that the hospital was otherwise in good order?	• OPEQ^b ^(Standard) • PEPC^c ^(Standard)	

24	Overall, was the help and treatment you received at the institution satisfactory?	• NORPEQ^a ^(Unidimensional)	X

25	Overall, what benefit have you had from the care at the institution?	• POPEQ^f ^(Outcome) • CAMHS^d ^(Outcome)	X

26	Do you believe that you were in any way given the wrong treatment (according to your own judgment)?	• NORPEQ^a^, OPEQ^b^, PEPC^c^. Single item increasingly used for comparison with administrative data	X

^a^: Nordic Patient Experiences Questionnaire [13]

^b^: Outpatient Experiences Questionnaire [14]

^c^: Parents Experiences of Paediatric Care [17]

^d^: Parent Assessment of Outpatient Child and Adolescent Mental Health Services [18]

^e^: Psychiatric Inpatient Experiences Questionnaire [15]

^f^: Psychiatric Outpatient Experiences Questionnaire [16]

^g^:The Norwegian Labour and Welfare Administration

Apart from the NORPEQ, all of the above instruments were developed separately and by following a similar development and validation process. (I) Preliminary questionnaires were constructed based on a literature review and open verbal interviews with users of the specific service. (II) Preliminary questionnaires were subjected to pre-testing by individual cognitive interviews with a small number of service users, who answered the questionnaire and then commented on the comprehensibility of the items and response options, as well as the relevance of the content and any missing topics. (III) A pilot test with subsequent assessment of the factor structure and psychometric properties completed the questionnaire development process. In 2008, NOKC had projects in progress to develop questionnaires for three more groups: cancer patients (including both out- and inpatient care), patients discharged from rehabilitation institutions, and patients in alcohol and substance dependency treatment.

### Construction of the questionnaire about experiences and importance

The above questionnaires had many single items in common; however, the factor structure varied. To ensure that all essential aspects were represented among the candidate items, we included one item from each dimension that was previously identified in each instrument. See Additional file 1 for an outline of the original questionnaires. We chose items with high factor loadings to maximise the response variation. The final number of generic items relating to patient experiences was 23. Some rephrasing was necessary for items with similar topics and dissimilar wording in the established instruments. Adjusting the wording of the items to fit the various user groups and care levels was a significant challenge. For example, the professionals responsible for the care of somatic inpatients are physicians, but for psychiatric outpatients, this may be a psychologist or a specialist nurse. We solved this problem by inserting an introductory paragraph before the items pertaining to the personnel: 'by "the clinicians" we mean: those who have had main responsibility for examinations and treatment. Most often these are physicians, but some receive care from psychologists or other health or social workers'; and 'By "the other staff" we mean: In hospital wards: the nursing staff or the milieu therapists/staff. In outpatient clinics or day care units: the staff you had contact with other than the clinician'. All items were expressed in second person singular. Next-of-kin to children were instructed to base their answers on their own perception of the care that was provided to their children. An English translation of the generic questionnaire about experiences and importance is included as Additional file 2.

The first three questions of the eight-page generic questionnaire were posed to identify the health care contact the respondents were evaluating during completion. All eight items from the brief NORPEQ were included. One item pertaining to both physicians and nursing personnel was divided in two in order to collect separate scores for the personnel groups. Another fourteen items were included from the other five questionnaires to cover dimensions not present in NORPEQ. Twenty-two items about patient experiences had a five-point response scale, which has been shown to be suitable for assessing patient experiences [20]: 1 = 'Not at all', 2 = 'To a small extent', 3 = 'To a moderate extent', 4 = 'To a large extent', and 5 = 'To a very large extent'. In addition, 'Not applicable' was a response option for 22 items. The item relating to waiting times used a four-point scale. Each item was followed directly by the question: 'How important was this to you?'. Because all items were included in the original questionnaires because of their previously demonstrated importance, we used a five-point response scale that allowed discrimination between the higher scores for importance in the generic questionnaire: 1 = 'Not important', 2 = 'A little important', 3='Important', 4 = 'Very important', and 5 = 'Of utmost importance' [21]. Six questions asked for individual background data such as gender, age (in 10-year intervals), self-rated health status, and level of education. Finally, the respondents were asked for their comments about whether there were important topics missing in the questionnaire, and if so, what these were.

After reviewing the contents of the single items and comparing them to the dimensions to which they belonged in the various original questionnaires, we classified the 23 items into nine dimensions: five items were related to clinician services; five to other staff services; two to information; two to involvement in decision-making; three to organisation and co-operation; one to accessibility; two to facilities at the institution; two to general outcome; and one to incorrect treatment.

The first version of the questionnaire was presented in individual cognitive interviews to 16 informants representing all the different target populations. Special attention was directed to potential problems with the items that had been changed to a less precise wording due to the diverse groups of respondents. We also collected information about comprehensibility and whether important topics were missing. The results from the verbal interviews led to minor adjustments in the questionnaire. The interviewees confirmed that the generic wording of the items worked as intended, as the frame of reference, for example of the imprecise expression "other staff", was sufficiently limited within the concrete situations that were to be evaluated.

#### Postal distribution and responses

The patient administrative system (PAS) was used to include patients to receive postal questionnaires. The primary inclusion criterion was being discharged from inpatient care or having had a consultation in outpatient care during November 2008 or being next-of-kin to a child in the same situation. For outpatients in psychiatric care we used an additional secondary criterion, as they were not included unless they also had had at least three consultations between the beginning of August and the end of November. The secondary inclusion criterion was introduced to avoid including persons that had scarce experience with the services such as patients only recently admitted to care or patients who had discontinued treatment after very short time. Treatments of short duration are frequent in somatic outpatient clinics; therefore, the secondary criterion was not implemented for these patients.

From previous surveys, we had information about response rates in the different groups (ranging from 35% of psychiatric inpatients to 54% of next-of-kin in paediatric care), and this information was used to estimate sufficient subsample sizes. To achieve an adequate size for each subsample, we adjusted the number of included patients according to the expected response rate. Groups with high patient turnover had a surplus of eligible individuals, and from these, we included a random sample. From groups with low turnover, we included all eligible individuals, recognising that 200 respondents for all groups would be unattainable within the study time frame.

The health care contact the respondents chose to evaluate did not necessarily correspond to the contact recorded in the PAS, by which their inclusion was induced. The hospital trust offered care in many locations and departments, and for individuals who did not respond until after the second reminder, ten weeks could have passed between being included and receiving the last reminder. Hence, respondents may have had several health care contacts in the meantime. Therefore, we used 'inclusion groups' based on information from the PAS when calculating response rates and comparing respondents to non-respondents, and in the analyses we used 'analysis groups' based on the contact identified by the respondents. Respondents who did not provide information about the type of contact they had described were associated with their inclusion group.

The questionnaires, along with an information letter and a postage-paid return envelope, were mailed by the health trust and returns were received at the NOKC. Reminders were mailed after two weeks, and if necessary, again after four more weeks to non- respondents. After mailing the second reminder, anonymous data were collected from the trust's PAS to be used for comparing respondents to non-respondents. The subsamples included by means of PAS information and contacted by mail as well as sample sizes and response rates, are shown in Table 2.

**Table 2:** Inclusion groups

Inclusion groups	Age (years)	Sample selection mode	Included (count)	Responded (count)	Response rate (%)
					
Adults					
Rehabilitation, outpatients	≥16	Consecutive, all included	242	122	50.4
Somatic, inpatients	≥16	Random	443	254	57.3
Somatic, outpatients	≥16	Random	381	219	57.5
Psychiatry, inpatients	≥18	Consecutive, all included	287	67	23.3
Psychiatry, outpatients^a^	≥18	Consecutive, all included	500	149	29.8
					
Children					
Somatic, inpatients	<16	Consecutive, all included	459	241	52.5
Psychiatry, outpatients^a^	<16	Consecutive, all included	500	173	34.6
					
Total					
			2812	1225^b,c^	43.6
					

Sample selection mode, sample size, and responses from postal distribution.

^a ^Additional inclusion criterion: Minimum three consultations between 1 August and 30 November 2008.

^b ^26 respondents among these individuals described a contact with alcohol and substance dependency care (9 inpatients and 17 outpatients), and are included accordingly in the analysis file.

^c ^The table is based on the file used for postal distribution and contains one case more than the analysis file. This difference cannot be accounted for as the files are never merged.

### Subsamples and response rates

The postal contacts yielded 1,225 returned questionnaires. The response rate in the postal survey was assessed in the inclusion groups and was 43.6%, varying considerably among the groups. Adult outpatients from somatic care had the highest response rate (57.3%) and adult psychiatric inpatients had the lowest (23.3%) (Table 2). Mean age for respondents was 51 years and for non-respondents 43 years (*t*-test, *p *< 0.001). The tendency to respond did not differ significantly between women and men (χ^2^-test).

The sizes of the analysis groups were comparable to their corresponding inclusion group sizes, but some small differences were observed. The largest differences were between adult in- and outpatients in somatic and psychiatric services because some respondents that were included as outpatients chose instead to report their inpatient experiences.

#### Personal distribution and responses

A previous review indicated that a better response rate may be achieved with personal distribution compared to postal distribution of questionnaires among patients in alcohol and substance dependency care [22]. Therefore, these patients were included consecutively and in person by the staff at the institutions. Questionnaires were delivered to inpatients at their discharge and to outpatients in connection with a consultation, and returned directly on site in a closed envelope. The distribution was anonymous, and sending reminders to non-respondents was not possible for this subsample. Patients were included from November 2008 to March 2009.

A form on which to record the personal questionnaire distribution was provided to the institutions in order to calculate response rates and assess potential non-response bias.

According to the respondents' reports, the analysis groups contained 52 inpatient and 74 outpatient respondents in the alcohol and substance dependency care group. Among these individuals, 26 were included by mail, as they were registered in the PAS with other health care contacts, mainly as outpatients in psychiatry or rehabilitation. The recordings of personal questionnaire distribution in the institutions were insufficient for calculating response rates. To assess the representativeness of this subsample, we obtained statistics about patients in these institutions in 2008 concerning age (in 10-year intervals) and gender. We used the median of the 10-year intervals to compare the respondent group with the population. Compared to the institutions' population of inpatients in 2008, the respondent group was older (mean age was 41.1 versus 45.3 years (*t*-test, *p *= 0.016)) and included more women (34.3 versus 41.2%), but the gender difference was not statistically significant (χ^2^-test). Compared to the institutions' population of outpatients, the respondent group was of the same age (mean age was 40.1 versus 41.1 years (*t*-test, n.s.)), and it had fewer women (35.9 versus 45.2%), but the gender difference was not statistically significant (χ^2^-test).

### Statistical analysis and criteria for identifying core items

The ten core items should be applicable for all user groups, address topics that are important in the eyes of the users, and cover different aspects of the health care experience.

The criteria used for selecting core items were as follows:

1. Applicability - the prevalence of responding "Not applicable" should be less than 20% in all the respondent groups. A proportion below 20% of item missing has been considered acceptable in previous studies [23]. This criterion implicated that items lacking relevance from the perspective of the respondents in any one of the groups should be excluded to avoid including redundant matter that many respondents would have no basis for evaluating. The remaining items could then be regarded as appropriate for the purpose of describing an aspect of patients' encounters with health care. Appropriateness is a central premise for an item to support good content validity [24-28].

2. Importance - the item should be important in the respondents' perspective (high group mean average). Items may be relevant, but still of minor importance. This criterion implicated consulting the target population to evaluate the importance of suggested items [26,28], and it followed other studies that have asked users to rate the importance of patient experience questions [2,9].

3. Comprehensiveness - the items should together cover a wide range of topics. It is widely accepted that patient experience is a multidimensional phenomenon. The number of items per dimension was limited to two to allow a variety of topics to be represented. This criterion ensured that the set of items would describe a broad scope of experiences providing the best possible content coverage within the limit of ten questions. Content representativeness is a central premise for good content validity [25,27,28].

To assess the association between the core items and global satisfaction, we used item 24 about overall satisfaction as the dependent variable in a regression model and the remaining nine core items as explanatory variables. To determine whether the core items that were selected using the three criteria were advantageous with regard to their ability to explain the variance in global satisfaction scores, we conducted ten multiple regressions with overall satisfaction as the dependent variable and nine explanatory variables that were randomly chosen among all candidate items in the generic questionnaire.

#### Total sample

For the analyses, we used a sample of 1,324 respondents, with subsample sizes from 52 (inpatients in dependency care) to 323 (adult somatic inpatients).

#### Approval

According to the joint body of the Norwegian Regional Committees for Medical and Health Research Ethics, approval is not required for quality assurance and evaluation projects that are part of the health services activities, even if the projects are carried out with scientific methods and aim to generate knowledge for potential publication, provided that the projects do not include changes in ordinary clinical practice. Also, ethical approval is not required if the data are anonymous [29]. The present study included no changes in clinical practice and the collected data were never linked to person-identifying information.

The health care trust notified The Data Inspectorate about the project (Notification no. 34106) and the responsibility for privacy protection in accordance with regulations was executed by a specific position at the trust.

The included individuals were informed that participation was voluntary and assured of confidentiality.

## Results

### Generic core items

The prevalence of "Not applicable" responses varied between the analysis groups and the different items—from zero to 58.1% (see Additional files 3 and 4). In general, outpatients judged the 'other staff' and the institutions' co-operation with external service providers to be less relevant. The first criterion left 13 items for further consideration. This conclusion was the same irrespective of whether the per cent "Not applicable" was calculated using the subsample n or the number of valid responses in the subsample as the denominator.

Importance scores were calculated as the average of group means. We tried alternative methods for producing the importance ranking (for example, the percentage of respondents ticking the highest possible score), and although the items' position in the ranking varied depending on the method, it did not vary to an extent that it altered the final set of items.

The 13 remaining items included all five items about the clinicians, and in accordance with the comprehensiveness criterion, only the two with the highest importance scores were selected as core items. Hence, the application of the stated criteria produced a set of ten generic core items (Table 3).

**Table 3:** Core items

**Item No**.	Item text
	
4	Did the *clinicians*^*a *^talk to you in a way that was easy to understand?
5	Do you have confidence in the *clinicians'*^*a *^professional skills?
15	Did you get sufficient information about your diagnosis/afflictions?
16	Did you perceive the treatment as adapted to your situation?
17	Were you involved in decisions regarding your treatment?
18	Did you perceive the institution's work as well organised?
21	Did you have to wait before you were admitted for services at the institution?
24	Overall, was the help and treatment you received at the institution satisfactory?
25	Overall, what benefit have you had from the care at the institution?
26	Do you believe that you were in any way given incorrect treatment (according to your own judgment)?

^a ^Note in the questionnaire: By *'the clinicians' *we mean: Those who have had main responsibility for examinations and treatment. Most often these are physicians, but some receive care from psychologists or other health or social workers.

The proportion of missing answers to the core items among the 1,324 respondents varied from 0.8% (item 4) to 4.2% (item 25) (see Additional file 4). The ceiling effect in the scores from the 1,324 respondents varied between the core items—from 13.4% (item 17) to 67.4% (item 26), and the mean ceiling effect between the ten items was 29.5% (data not shown). There were differences between the groups' scores for all the items regarding both health care experiences and importance (see Additional file 5).

### Core items and general satisfaction

The adjusted R^2 ^for the model with nine core items regressed on item 24 showed that 70% of the variation in the general satisfaction score could be explained by the scores on the other nine core items in the total sample, ranging from 43% (alcohol and substance dependency care, inpatients) to 81% (psychiatric care, inpatients) in the subsamples. The direct effect of item 17 about involvement in decision-making was not statistically significant, and the item about general outcome had the strongest effect. In the ten models with randomly chosen explanatory variables, the explained variance in the general satisfaction score varied from 57% to 68% (mean 64%) in the total sample.

## Discussion

The study identified ten generic core items covering major dimensions of experiences that patients across a range of specialist health care services report to be important for user groups. To our knowledge, this is the first generic, short questionnaire for collecting data about user experiences across different types of services (inpatient, outpatient), patient groups (somatic, psychiatry, addiction, rehabilitation), and user groups (patients, next-of-kin to children).

The low proportion of missing answers indicates good acceptability of the generic questionnaire, and means that the survey findings are relevant to large proportions of the target populations. The ceiling effect varied among the items. A high ceiling effect is a common feature in measures of patient experiences [30], and reduces the instruments' ability to describe differences through time or between units or institutions. This is a small concern when the instrument is used for large-scale performance monitoring, but may be a weakness if it is to be included in small-scale quality improvement documentation. However, the ceiling effect in this study is a direct consequence of the ceiling effects in the questionnaires we built upon, so it is not a specific concern for the Generic Short Patient Experiences Questionnaire (GS-PEQ).

The present project differed from traditional psychometric development and assessment, in which phenomena that are central for specific groups are identified and then operationalized with multi-item scales for reliable measurement. The present short, generic questionnaire cannot provide information with comparable specificity [31]. The purpose of conducting patient evaluation varies and the methods must be adapted to the context in question [32]. The GS-PEQ is a useful alternative to a single global item or lengthier questionnaires when used in large samples to meet the strategic level management's need for quality monitoring Caution has been raised regarding the use of single global items to collect patients' evaluation [33], and lengthier questionnaires increase the use of resources.

If the purpose of patient surveys is to inform decision-making or evaluate the services at the operational management level, the core items alone will be lacking in specificity, and hence, be less useful [34]. For such purposes, the core items should be used along with tools that specifically address the context in question.

To obtain a high response rate, it is recommended to ensure that the questionnaire content is relevant to potential respondents [11]. Therefore, one argument against a generic format would be that it could have a negative impact on the response rate. Peytremann-Bridevaux et al. [35] compared one generic and two psychiatric—specific patient satisfaction questionnaires in a sample of psychiatric patients. The results indicated that none of the instruments was superior to the others regarding the measurement properties-including response rates—and the patients' evaluation of the questionnaires.

The ten items in the GS-PEQ were rated as having high importance and relevance by respondents in this study. The agreement between importance and relevance suggests that the selected core items reflect general wishes pertaining to all types of health care; a positive outcome from health care enacted by clinicians that are competent and take their patients seriously within a well organised service. However; this implies a limitation regarding more specific perspectives. For example, the question regarding preparation for the time after the treatment was finished was excluded because the proportion "not applicable" score was more than 20 percent in three groups. Among respondents in inpatient substance and alcohol dependency care only four percent found this question "not applicable" and also ranked it fourth in importance. Less than one percent of the respondents who reported experiences from inpatient pediatric care found the question regarding other staff's skills "not applicable" and ranked it ninth in importance. Hence; in surveys of specific groups, one should consider including items that are rated as important by that specific group, but are not included among the core items due to low applicability for any of the other groups (see Additional files 4 and 5).

The low response rate—despite using two reminders-is a potential limitation in the study. It is important to assess the influence of non-response bias [36]; however, previous studies suggest that the non-response impact is relatively small [37]. Potential non-response bias has been studied in three of the present target populations in Norway: adult somatic inpatients [38], next-of-kin to children in outpatient psychiatric care [39], and adult psychiatric outpatients [40]. These studies showed that there are only minor differences between the postal respondents and the postal non-respondents who provided answers through follow-up telephone interviews. It is not known whether these findings can be generalised to populations where such studies have not been conducted. For these populations, the present results should be interpreted with caution.

The simultaneous inclusion of nine user groups is a strength of the present study. However, even if the health trust in question serves both local and regional functions, it is still a one-site study and we do not know to what degree the samples represent users in Norwegian hospitals in general. In addition, we cannot exclude the possibility that further subsample division, e.g., into condition specific groups, might have shown differences between respondents with different conditions. A recent study has shown that patients' priorities varied between five patient groups that were all are categorised as somatic in the current study [33].

The criteria for selecting the ten core items were used to secure content coverage. As a complement to this method, regression analysis was conducted in which the single item about general satisfaction (item 24) was used as the dependent variable and the remaining items as predictors for this variable [33,41]. The fact that the selected items could account for a large proportion of the variance in global satisfaction gives additional evidence for the claim that the GS-PEQ covers relevant aspects of health care experiences across the included health services. Moreover, this regression model was compared to ten other models with random selection of predictor variables across the 23 piloted items. The differences between the random models and the model with the selected items were small, but consistently in favour of the model with the selected core items. Of particular concern is the low proportion of explained variance for inpatients in dependency care compared to other groups, which may indicate that important variables are missing for this group. However, our instruction to the respondents to report missing topics in both the verbal interviews and in the present survey did not provide new topics, leaving a substantiated assumption that all topics of importance were covered.

Test-retest reliability and response rates for the GS-PEQ could not be calculated for the data collected in the present survey. The effect of different distribution modes should be studied such as when or where the responses are given (i.e., at home on paper or on site on touch-screens).

Single items are normally less reliable than scales [42], meaning that larger sample sizes are needed for single items to achieve a similar level of score precision. Further research should assess sample sizes needed to achieve satisfactory reliability levels for items in the GS-PEQ, in addition to data quality issues and psychometric properties in studies using GS-PEQ as a standalone tool or alongside other measures. Further research is also needed to examine the usefulness of comparing results between different health care services. For example, the relatively low scores for many of the experiences for psychiatric inpatients may be a true reflection of service quality at this specific hospital trust. The low scores from psychiatric patients may also be explained by their health condition, as was shown in a previous study in which there were similar differences when comparing this group to others [43].

## Conclusions

The GS-PEQ is a comprehensive and efficient set of items covering important aspects of user experiences across a range of specialist health care services. The GS-PEQ could be used alone or with other instruments in quality measurement activities at different levels or in research, but the relevance of GS-PEQ for other health care settings and countries requires further evaluation.

## Competing interests

The Norwegian Knowledge Centre for the Health Services is a non-commercial public organisation that regularly conducts national surveys for varying target populations receiving or delivering health care.

## Authors' contributions

ISS planned the paper with OAB, RVO, HII, and GB, co-operated with the health trust in data collection, carried out the statistical analysis, and drafted the paper. All authors contributed with critical revisions of the draft and all authors read and approved the final manuscript.

## Pre-publication history

The pre-publication history for this paper can be accessed here:

http://www.biomedcentral.com/1472-6963/11/88/prepub

## Supplementary Material

Additional file 1 Outline of the six original and source questionnaires. Dimension count, topics, and number of items Click here for file

Additional file 2 Generic questionnaire about experiences and importance Click here for file

Additional file 3 **Table displaying generic items ordered by importance score and with highest prevalence of "Not applicable" in any group shown. Core items in bold**. Click here for file

Additional file 4 Table displaying item per cent missing and 'Not applicable' responses per analysis group Click here for file

Additional file 5 Table displaying item scores on user experiences and importance per analysis group Click here for file
