# Supplementary material for: The Generic Short Patient Experiences Questionnaire (GS-PEQ): identification of core items from a survey in Norway
Source: BMC Health Serv Res. 2011 Apr 21;11:88. doi: 10.1186/1472-6963-11-88 (PMC3111343; doi:10.1186/1472-6963-11-88)
Supplement: Additional file 1 — Outline of the six original and source questionnaires. Dimension count, topics, and number of items [file 1472-6963-11-88-S1.PDF]

# Additional file 1

## The six original and source questionnaires. Dimension count, topics, and number of items.

| Questionnaire | Dimensions count | Dimension topics; item count | Source of generic items (No)[b] |
|---|---|---|---|
| Nordic patients' experiences questionnaire | 1[a] | Doctors understandable | **4** |
| | | Doctors' professional skills | **5** |
| | | Nurses' professional skills | 9 |
| | | Nursing care | 10 |
| | | Doctors and nurses interested in problem | 7, 12 |
| | | Information about tests | 14 |
| | | General satisfaction | **24** |
| | | Incorrect treatment | **26** |
| Outpatients' experiences questionnaire | 3[a] | Communication; 6 items | **4**, **5**, 6, 7, 8, |
| | | Organisation; 4 items | **18** |
| | | Accessibility; 2 items | **21** |
| Psychiatric inpatients' experiences questionnaire | 1[a] | Preparation for discharge | 19, 20 |
| | | Enough time with therapists/personnel | 8, 13 |
| | | Therapists/personnel understood situation | 7, 12 |
| | | Therapists/personnel listened | 7, 12 |
| | | Treatment suitability | **16** |
| | | Involvement in treatment decisions | **17** |
| | | Information about treatment options | **15** |
| | | Information about condition/diagnosis | **15** |
| Psychiatric outpatients' experiences questionnaire | 3[a] | Outcome; 3 items | 25 |
| | | Clinician interaction; 6 items | 7, 8, **16**, **17** |
| | | Information; 2 items | **15** |
| Parents' experiences of pediatric care | 6[a] | Nursing services; 7 items | 9, 11, 12 |
| | | Doctor services; 5 items | **4**, **5**, 6, 7 |
| | | Organisation; 4 items | **18** |
| | | Information - examinations and tests; 2 items | 14 |
| | | Information - discharge; 3 items | 19 |
| | | Hospital facilities; 4 items | 22, 23 |
| Parents' assessment of outpatient child and adolescent mental health services | 3[a] | Clinician interaction; 8 items | **4**, 6, 7, 8 |
| | | Treatment outcome; 2 items | **25** |
| | | Information and involvement; 4 items | **15**, **17** |

[a] Established by psychometric assessment

[b] Subsequently selected core items are in bold
