# Supplementary material for: The Generic Short Patient Experiences Questionnaire (GS-PEQ): identification of core items from a survey in Norway
Source: BMC Health Serv Res. 2011 Apr 21;11:88. doi: 10.1186/1472-6963-11-88 (PMC3111343; doi:10.1186/1472-6963-11-88)
Supplement: Additional file 2 — Generic questionnaire about experiences and importance [file 1472-6963-11-88-S2.PDF]

Each question is about:
a - Your experiences during the contact you reported on the previous page.
b - How important the question's topic was to you.
If a question does not apply to you, please use the "Not applicable" option.

About the clinicians

By "the clinicians" we mean those who have had main responsibility for examinations and treatments. Most often these are physicians, but some receive care from psychologists or other health or social workers.

Questions 9 - 13 are about the other staff.

4a. Did the clinicians talk to you in a way that was easy to understand?

Not at all	To a small extent	To a moderate extent	To a large extent	To a very large extent	Not applicable
☐	☐	☐	☐	☐	☐

4b. How important was this to you?

Not important	A little important	Important	Very important	Of utmost importance
☐	☐	☐	☐	☐

5a. Do you have confidence in the clinicians' professional competence?

Not at all	To a small extent	To a moderate extent	To a large extent	To a very large extent	Not applicable
☐	☐	☐	☐	☐	☐

5b. How important was this to you?

Not important	A little important	Important	Very important	Of utmost importance
☐	☐	☐	☐	☐

6a. To what degree did you perceive that the clinicians cared about you?

Not at all	To a small extent	To a moderate extent	To a large extent	To a very large extent	Not applicable
☐	☐	☐	☐	☐	☐

6b. How important was this to you?

Not important	A little important	Important	Very important	Of utmost importance
☐	☐	☐	☐	☐

7a. Did you perceive the clinicians to be interested in your description of your situation?

Not at all	To a small extent	To a moderate extent	To a large extent	To a very large extent	Not applicable
☐	☐	☐	☐	☐	☐

7b. How important was this to you?

Not important	A little important	Important	Very important	Of utmost importance
☐	☐	☐	☐	☐

8a. Did you get enough time to talk and interact with the clinicians?

Not at all	To a small extent	To a moderate extent	To a large extent	To a very large extent	Not applicable
☐	☐	☐	☐	☐	☐

8b. How important was this to you?

Not important	A little important	Important	Very important	Of utmost importance
☐	☐	☐	☐	☐

About the other staff

By "the other staff" we mean
In hospital wards: the nursing staff or milieu therapists/staff
In outpatient clinics or day care units: the staff you had contact with other than the clinician

9a. Did the other staff talk to you in a way that was easy to understand?

Not at all	To a small extent	To a moderate extent	To a large extent	To a very large extent	Not appli-cable
☐	☐	☐	☐	☐	☐

9b. How important was this to you?

Not impor-tant	A little impor-tant	Impor-tant	Very impor-tant	Of utmost impor-tance
☐	☐	☐	☐	☐

10a. Do you have confidence in the other staff's professional skills?

Not at all	To a small extent	To a moderate extent	To a large extent	To a very large extent	Not appli-cable
☐	☐	☐	☐	☐	☐

10b. How important was this to you?

Not impor-tant	A little impor-tant	Impor-tant	Very impor-tant	Of utmost impor-tance
☐	☐	☐	☐	☐

11a. To what degree did you perceive that the other staff cared about you?

Not at all	To a small extent	To a moderate extent	To a large extent	To a very large extent	Not appli-cable
☐	☐	☐	☐	☐	☐

11b. How important was this to you?

Not impor-tant	A little impor-tant	Impor-tant	Very impor-tant	Of utmost impor-tance
☐	☐	☐	☐	☐

12a. Did you perceive the other staff to be interes-ted in your description of your situation?

Not at all	To a small extent	To a moderate extent	To a large extent	To a very large extent	Not appli-cable
☐	☐	☐	☐	☐	☐

12b. How important was this to you?

Not impor-tant	A little impor-tant	Impor-tant	Very impor-tant	Of utmost impor-tance
☐	☐	☐	☐	☐

13a. Did you get enough time to talk and interact with the other staff?

Not at all	To a small extent	To a moderate extent	To a large extent	To a very large extent	Not appli-cable
☐	☐	☐	☐	☐	☐

13b. How important was this to you?

Not impor-tant	A little impor-tant	Impor-tant	Very impor-tant	Of utmost impor-tance
☐	☐	☐	☐	☐

About information and co-operation

14a. Were you told as much as you considered necessary about how tests or examinations would be carried out?

Not at all	To a small extent	To a moderate extent	To a large extent	To a very large extent	Not appli-cable
☐	☐	☐	☐	☐	☐

14b. How important was this to you?

Not impor-tant	A little impor-tant	Impor-tant	Very impor-tant	Of utmost impor-tance
☐	☐	☐	☐	☐

15a. Did you get sufficient information about your diagnosis/your afflictions?

Not at all	To a small extent	To a moderate extent	To a large extent	To a very large extent	Not appli-cable
☐	☐	☐	☐	☐	☐

15b. How important was this to you?

Not impor-tant	A little impor-tant	Impor-tant	Very impor-tant	Of utmost impor-tance
☐	☐	☐	☐	☐

16a. Did you perceive the treatment you received as suited to your situation?

Not at all	To a small extent	To a moderate extent	To a large extent	To a very large extent	Not appli-cable
☐	☐	☐	☐	☐	☐

16b. How important was this to you?

Not impor-tant	A little impor-tant	Impor-tant	Very impor-tant	Of utmost impor-tance
☐	☐	☐	☐	☐

17a. Were you involved in any decisions regarding your treatment?

Not at all	To a small extent	To a moderate extent	To a large extent	To a very large extent	Not appli-cable
☐	☐	☐	☐	☐	☐

17b. How important was this to you?

Not impor-tant	A little impor-tant	Impor-tant	Very impor-tant	Of utmost impor-tance
☐	☐	☐	☐	☐

18a. Did you perceive the institution's work as well organised?

Not at all	To a small extent	To a moderate extent	To a large extent	To a very large extent	Not appli-cable
☐	☐	☐	☐	☐	☐

18b. How important was this to you?

Not impor-tant	A little impor-tant	Impor-tant	Very impor-tant	Of utmost impor-tance
☐	☐	☐	☐	☐

19a. Did you perceive that the institution prepared you for the time after the treatment was finished?

Not at all	To a small extent	To a moderate extent	To a large extent	To a very large extent	Not appli-cable
☐	☐	☐	☐	☐	☐

19b. How important was this to you?

Not impor-tant	A little impor-tant	Impor-tant	Very impor-tant	Of utmost impor-tance
☐	☐	☐	☐	☐

20a. Do you find that the institution has co-operated well with other public services (e.g. your GP, NAV*, or district nurse)?

*:The Norwegian Labour and Welfare Administration

Not at all	To a small extent	To a moderate extent	To a large extent	To a very large extent	Not appli-cable
☐	☐	☐	☐	☐	☐

20b. How important was this to you?

Not impor-tant	A little impor-tant	Impor-tant	Very impor-tant	Of utmost impor-tance
☐	☐	☐	☐	☐

Other questions

21a. Did you have to wait before you were admitted for services at the institution?

No	Yes, but not long	Yes, quite long	Yes, much too long
☐	☐	☐	☐

21b. How important was this to you?

Not impor-tant	A little impor-tant	Impor-tant	Very impor-tant	Of utmost impor-tance
☐	☐	☐	☐	☐

22a. Did you get the impression that the hospital equipment was in good order?

Not at all	To a small extent	To a moderate extent	To a large extent	To a very large extent	Not appli-cable
☐	☐	☐	☐	☐	☐

22b. How important was this to you?

Not impor-tant	A little impor-tant	Impor-tant	Very impor-tant	Of utmost impor-tance
☐	☐	☐	☐	☐

23a. Did you get the impression that the hospital was otherwise in good order?

Not at all	To a small extent	To a moderate extent	To a large extent	To a very large extent	Not appli-cable
☐	☐	☐	☐	☐	☐

23b. How important was this to you?

Not impor-tant	A little impor-tant	Impor-tant	Very impor-tant	Of utmost impor-tance
☐	☐	☐	☐	☐

24a. Overall, was the help and treatment you received at the institution satisfactory?

Not at all	To a small extent	To a moderate extent	To a large extent	To a very large extent	Not appli-cable
☐	☐	☐	☐	☐	☐

24b. How important was this to you?

Not impor-tant	A little impor-tant	Impor-tant	Very impor-tant	Of utmost impor-tance
☐	☐	☐	☐	☐

25a. Overall, what benefit have you had from the care at the institution?

No benefit	Small benefit	Some benefit	Great benefit	Huge benefit	Not ap-plicable
☐	☐	☐	☐	☐	☐

25b. How important was this to you?

Not impor-tant	A little impor-tant	Impor-tant	Very impor-tant	Of utmost impor-tance
☐	☐	☐	☐	☐

26a. Do you believe that you were in any way given the wrong treatment (according to your own judgment)?

Not at all	To a small extent	To a moderate extent	To a large extent	To a very large extent	Not appli-cable
☐	☐	☐	☐	☐	☐

26b. How important was this to you?

Not impor-tant	A little impor-tant	Impor-tant	Very impor-tant	Of utmost impor-tance
☐	☐	☐	☐	☐
