# Supplementary material for: The Generic Short Patient Experiences Questionnaire (GS-PEQ): identification of core items from a survey in Norway
Source: BMC Health Serv Res. 2011 Apr 21;11:88. doi: 10.1186/1472-6963-11-88 (PMC3111343; doi:10.1186/1472-6963-11-88)
Supplement: Additional file 3 — Table displaying generic items ordered by importance score and with highest prevalence of "Not applicable" in any group shown. Core items in bold. [file 1472-6963-11-88-S3.PDF]

Additional file 3

Generic items ordered by importance score and with highest prevalence of "Not applicable" in any group shown. Core items in bold.

Item No.	Item text	Importance[a]	Max % 'Not applicable' by groups[b]
**25**	**Overall, what benefit have you had from the care at the institution?**	4.23	12.1
**5**	**Do you have confidence in the *clinicians'*[c] professional skills?**	4.23	1.04
**24**	**Overall, was the help and treatment you received at the institution satisfactory?**	4.2	5.41
**26**	**Do you believe that you were in any way given incorrect treatment (according to your own judgement)?**	4.13	17.83
**4**	**Did the *clinicians*[c] talk to you in a way that was easy to understand?**	4.13	1.12
**16**	**Did you perceive the treatment as adapted to your situation?**	4.13	10.19
**15**	**Did you get sufficient information about your diagnosis/ afflictions?**	4.02	10.11
7	Did you perceive the *clinicians*[c] to be interested in your description of your situation?	4.01	3.32
8	Did you get enough time to talk and interact with the *clinicians*[c]?	3.94	1.27
6	To what degree did you perceive that the *clinicians*[c] cared about you?	3.93	0.83
**21**	**Did you have to wait before you were admitted for services at the institution?**	3.85	-
14	Were you told as much as you considered necessary about how tests or examinations would be carried out?	3.83	33.78
19	Did you perceive that the institution prepared you for the time after the treatment was finished?	3.83	51.69
**17**	**Were you involved in decisions regarding your treatment?**	3.76	15.92
**18**	**Did you perceive the institution's work as well organised?**	3.73	12.36
10	Do you have confidence in *the other staff's*[d] professional skills?	3.73	28.09
9	Did *the other staff*[d] talk to you in a way that was easy to understand?	3.69	22.47
20	Did you find that the institution has co-operated well with other public services (e.g., your GP, NAV[e], or home nursing care)?	3.63	40.54
11	To what degree did you perceive that *the other staff*[d] cared about you?	3.55	43.24
22	Did you get the impression that the hospital equipment was in good order?	3.48	49.44
12	Did you perceive *the other staff*[d] to be interested in your description of your situation?	3.4	58.11
23	Did you get the impression that the hospital otherwise was in good order?	3.39	20.22
13	Did you get enough time to talk and interact with *the other staff*[d]?	3.3	50.56

[a] Average of nine group means.

[b] Percentage in the one single group with the highest prevalence.

[c] Note in the questionnaire: By 'the clinicians' we mean: Those who have had main responsibility for examinations and treatment. Most often these are physicians, but some receive care from psychologists or other health or social workers.

[d] Note in the questionnaire: By 'the other staff' we mean: In hospital wards → the nursing staff or the milieu therapists/staff. In outpatient clinics or day units → the staff you had contact with other than the clinicians.

[e] The Norwegian Labour and Welfare Administration.
