# Supplementary material for: The Generic Short Patient Experiences Questionnaire (GS-PEQ): identification of core items from a survey in Norway
Source: BMC Health Serv Res. 2011 Apr 21;11:88. doi: 10.1186/1472-6963-11-88 (PMC3111343; doi:10.1186/1472-6963-11-88)
Supplement: Additional file 4 — Table displaying item per cent missing and 'Not applicable' responses per analysis group [file 1472-6963-11-88-S4.PDF]

# Additional file 4

Item per cent missing and ‘Not applicable’ responses per analysis group

| | Postal questionnaire distribution | | | | | | | | | | | | | | Personal questionnaire distribution | | | |
|---|---|---|---|---|---|---|---|---|---|---|---|---|---|---|---|---|---|---|
| | Adults | | | | | | | | | | Next-of-kin to children | | | | Adults | | | |
| Item. number | Rehabilitation, outpat. (N=105) | | Somatic, inpat. (N=323) | | Somatic, outpat. (N=187) | | Psychiatry, inpat. (N=96) | | Psychiatry, outpat. (N=89) | | Somatic, inpat. (N=241) | | Psychiatry, outpat. (N=157) | | Dependence, inpat. (N=52) | | Dependence, outpat. (N=74) | |
| | % Mis. | % NA | % Mis. | % NA | % Mis. | % NA | % Mis. | % NA | % Mis. | % NA | % Mis. | % NA | % Mis. | % NA | % Mis. | % NA | % Mis. | % NA |
| 4 | 0.95 | 0.00 | 0.31 | 0.62 | 2.14 | 0.00 | 1.04 | 0.00 | 0.00 | 1.12 | 0.41 | 0.00 | 1.91 | 0.00 | 0.00 | 0.00 | 0.00 | 0.00 |
| 5 | 0.95 | 0.00 | 0.93 | 0.00 | 2.14 | 0.00 | 0.00 | 1.04 | 0.00 | 0.00 | 0.83 | 0.00 | 1.27 | 0.00 | 1.92 | 0.00 | 0.00 | 0.00 |
| 6 | 1.90 | 0.00 | 0.62 | 0.31 | 2.14 | 0.00 | 0.00 | 0.00 | 0.00 | 0.00 | 1.24 | 0.83 | 0.64 | 0.00 | 0.00 | 0.00 | 0.00 | 0.00 |
| 7 | 1.90 | 0.95 | 1.24 | 0.62 | 2.14 | 1.07 | 0.00 | 0.00 | 0.00 | 0.00 | 0.41 | 3.32 | 1.27 | 1.91 | 1.92 | 0.00 | 0.00 | 0.00 |
| 8 | 0.95 | 0.00 | 0.93 | 0.62 | 2.14 | 0.00 | 0.00 | 0.00 | 1.12 | 0.00 | 0.41 | 0.41 | 1.27 | 1.27 | 1.92 | 0.00 | 0.00 | 0.00 |
| 9 | 0.95 | 2.86 | 0.93 | 1.24 | 3.21 | 5.88 | 1.04 | 0.00 | 1.12 | 22.47 | 0.83 | 1.66 | 2.55 | 19.75 | 1.92 | 1.92 | 4.05 | 17.57 |
| 10 | 1.90 | 3.81 | 1.24 | 0.31 | 3.74 | 6.95 | 2.08 | 0.00 | 2.25 | 28.09 | 1.24 | 0.83 | 2.55 | 18.47 | 3.85 | 0.00 | 5.41 | 24.32 |
| 11 | 4.76 | 3.81 | 0.93 | 0.62 | 3.74 | 9.63 | 2.08 | 0.00 | 0.00 | 30.34 | 0.83 | 1.66 | 3.82 | 22.29 | 1.92 | 0.00 | 4.05 | 43.24 |
| 12 | 3.81 | 9.52 | 2.17 | 3.41 | 5.88 | 17.65 | 2.08 | 0.00 | 0.00 | 47.19 | 0.83 | 4.15 | 3.18 | 29.30 | 1.92 | 1.92 | 5.41 | 58.11 |
| 13 | 4.76 | 7.62 | 1.55 | 2.79 | 4.28 | 18.72 | 2.08 | 1.04 | 0.00 | 50.56 | 0.83 | 2.07 | 3.82 | 32.48 | 1.92 | 1.92 | 4.05 | 40.54 |
| 14 | 3.81 | 2.86 | 2.17 | 1.24 | 1.60 | 2.14 | 3.13 | 10.42 | 1.12 | 24.72 | 0.83 | 1.24 | 2.55 | 8.28 | 3.85 | 1.92 | 0.00 | 33.78 |
| 15 | 1.90 | 0.00 | 0.93 | 1.86 | 1.60 | 5.35 | 0.00 | 5.21 | 1.12 | 10.11 | 0.83 | 3.73 | 2.55 | 8.92 | 1.92 | 5.77 | 0.00 | 6.76 |
| 16 | 2.86 | 3.81 | 1.24 | 1.55 | 3.74 | 6.42 | 0.00 | 1.04 | 0.00 | 5.62 | 0.83 | 5.39 | 3.18 | 10.19 | 1.92 | 1.92 | 1.35 | 4.05 |
| 17 | 3.81 | 3.81 | 1.24 | 6.50 | 3.74 | 12.30 | 0.00 | 2.08 | 0.00 | 13.48 | 1.66 | 11.20 | 2.55 | 15.92 | 1.92 | 1.92 | 0.00 | 5.41 |
| 18 | 1.90 | 0.95 | 0.93 | 1.86 | 3.21 | 3.21 | 1.04 | 1.04 | 0.00 | 12.36 | 2.07 | 2.07 | 2.55 | 7.01 | 1.92 | 0.00 | 0.00 | 6.76 |
| 19 | 5.71 | 12.38 | 2.48 | 6.50 | 4.81 | 24.06 | 2.08 | 6.25 | 1.12 | 51.69 | 2.90 | 14.94 | 3.18 | 31.85 | 5.77 | 3.85 | 2.70 | 18.92 |
| 20 | 5.71 | 18.10 | 4.33 | 19.81 | 6.95 | 26.74 | 2.08 | 4.17 | 0.00 | 26.97 | 3.73 | 34.02 | 2.55 | 21.02 | 7.69 | 7.69 | 1.35 | 40.54 |
| 21 | 3.81 | - | 5.88 | - | 3.21 | - | 2.08 | - | 1.12 | - | 2.90 | - | 7.64 | - | 1.92 | - | 4.05 | - |
| 22 | 5.71 | 7.62 | 4.95 | 4.33 | 4.81 | 6.95 | 3.13 | 10.42 | 0.00 | 49.44 | 1.66 | 3.32 | 5.10 | 28.66 | 3.85 | 3.85 | 8.11 | 25.68 |
| 23 | 6.67 | 3.81 | 4.64 | 3.41 | 5.35 | 8.02 | 3.13 | 3.13 | 0.00 | 20.22 | 2.07 | 2.07 | 5.73 | 12.74 | 1.92 | 1.92 | 4.05 | 13.51 |
| 24 | 7.62 | 0.95 | 3.41 | 1.55 | 3.74 | 1.60 | 2.08 | 0.00 | 1.12 | 2.25 | 2.90 | 0.41 | 5.10 | 4.46 | 1.92 | 0.00 | 2.70 | 5.41 |
| 25 | 7.62 | 4.76 | 4.02 | 4.33 | 5.35 | 7.49 | 2.08 | 0.00 | 3.37 | 2.25 | 3.32 | 5.81 | 5.73 | 12.10 | 1.92 | 1.92 | 2.70 | 5.41 |
| 26 | 5.71 | 6.67 | 4.95 | 6.19 | 6.95 | 9.09 | 1.04 | 4.17 | 1.12 | 8.99 | 3.32 | 11.20 | 4.46 | 17.83 | 1.92 | 3.85 | 2.70 | 9.46 |
| 27 | 4.76 | 2.86 | 4.02 | 3.10 | 5.35 | 8.02 | 2.08 | 4.17 | 1.12 | 5.62 | 3.32 | 2.49 | 5.10 | 10.19 | 1.92 | 0.00 | 2.70 | 5.41 |
