# Supplementary material for: The Generic Short Patient Experiences Questionnaire (GS-PEQ): identification of core items from a survey in Norway
Source: BMC Health Serv Res. 2011 Apr 21;11:88. doi: 10.1186/1472-6963-11-88 (PMC3111343; doi:10.1186/1472-6963-11-88)
Supplement: Additional file 5 — Table displaying item scores on user experiences and importance per analysis group [file 1472-6963-11-88-S5.PDF]

Additional file 5

Item scores on user experiences and importance per analysis group

	Postal questionnaire distribution														Personal questionnaire distribution			
	Adults										Next-of-kin to children				Adults			
Item number	Rehabilitation, outpat. (N=105)		Somatic, inpat. (N=323)		Somatic, outpat. (N=187)		Psychiatry, inpat. (N=96)		Psychiatry, outpat. (N=89)		Somatic, inpat. (N=241)		Psychiatry, outpat. (N=157)		Dependence, inpat. (N=52)		Dependence, outpat. (N=74)	
	Exper.	Import.	Exper.	Import.	Exper.	Import.	Exper.	Import.	Exper.	Import.	Exper.	Import.	Exper.	Import.	Exper.	Import.	Exper.	Import.
4	4.34	4.08	4.16	4.14	4.20	3.99	3.72	3.84	4.38	4.20	4.28	4.34	4.23	4.27	4.13	4.06	4.08	4.28
5	4.13	4.17	4.32	4.30	4.30	4.20	3.46	3.87	4.13	4.28	4.26	4.51	4.12	4.35	3.96	4.06	4.00	4.34
6	3.94	3.88	4.01	4.03	4.04	3.96	3.31	3.93	4.04	4.16	3.80	4.00	3.94	3.95	4.04	3.88	3.50	3.55
7	3.91	4.05	3.79	3.92	3.92	3.94	3.28	3.88	4.16	4.33	3.78	3.92	4.08	4.16	4.02	3.78	3.66	4.14
8	3.90	3.92	3.52	3.91	3.87	3.91	3.10	3.77	3.98	4.07	3.50	3.98	3.71	4.09	3.69	3.80	3.78	3.97
9	4.16	3.67	3.99	3.82	4.11	3.64	3.72	3.78	4.01	3.36	4.17	4.02	4.04	3.84	3.84	3.77	4.48	3.31
10	4.08	3.70	4.07	3.90	4.09	3.75	3.26	3.74	3.77	3.42	3.94	4.07	3.94	3.94	3.76	3.72	4.23	3.36
11	3.74	3.49	3.96	3.87	4.01	3.60	3.45	3.86	3.52	3.42	3.73	3.79	3.69	3.55	3.82	3.64	3.51	2.71
12	3.59	3.52	3.62	3.59	3.70	3.42	3.19	3.76	3.32	3.20	3.58	3.62	3.53	3.50	3.66	3.52	3.59	2.48
13	3.52	3.39	3.36	3.50	3.56	3.27	3.28	3.64	3.20	2.85	3.44	3.52	3.45	3.41	3.48	3.38	3.93	2.77
14	3.80	3.73	3.77	3.83	3.92	3.91	3.05	3.70	3.62	3.83	3.81	4.15	3.81	4.06	3.31	3.49	3.96	3.81
15	3.58	4.13	3.67	4.01	3.82	4.12	2.92	3.90	3.51	3.91	3.70	4.20	3.57	4.16	3.40	3.74	3.62	4.04
16	3.68	4.22	3.96	4.10	4.04	4.09	3.02	4.00	3.80	4.23	3.87	4.21	3.71	4.15	3.78	3.82	3.47	4.33
17	3.37	3.67	2.84	3.36	3.21	3.67	2.85	3.93	3.83	3.90	2.86	3.43	3.52	3.89	3.34	3.76	3.81	4.26
18	4.00	3.73	3.70	3.71	3.70	3.67	3.13	3.75	3.78	3.61	3.40	3.80	3.69	3.88	3.67	3.55	3.35	3.91
19	3.51	3.90	3.13	3.70	3.35	3.79	2.83	3.79	3.14	3.64	3.16	3.71	3.09	3.94	3.09	4.04	3.12	3.95
20	3.29	3.69	3.01	3.51	3.27	3.63	2.83	3.52	3.58	3.84	3.10	3.41	3.30	3.95	2.86	3.79	2.47	3.31
21[a]	2.68	3.79	3.10	3.96	3.02	3.66	3.48	3.60	2.75	4.10	3.19	4.06	2.43	4.09	2.57	3.82	2.56	3.53
22	3.90	3.71	4.07	3.95	4.04	3.79	3.40	3.33	4.07	3.13	3.81	3.98	3.85	3.76	3.29	3.43	4.00	2.26
23	3.82	3.61	3.83	3.73	3.90	3.65	3.43	3.42	4.08	3.12	3.24	3.63	3.84	3.50	3.34	3.38	4.00	2.48
24	3.92	4.13	4.08	4.19	4.13	4.13	3.19	4.10	3.87	4.33	4.02	4.32	3.89	4.25	3.88	3.96	3.76	4.38
25	3.43	4.13	3.96	4.25	3.88	4.21	3.15	4.03	3.73	4.35	3.94	4.32	3.74	4.36	3.92	4.18	3.38	4.25
26	4.48	3.98	4.52	4.23	4.54	4.14	3.46	3.89	4.33	4.05	4.57	4.33	4.43	4.24	4.37	3.83	4.62	4.54
27	3.67	-	3.59	-	3.70	-	3.20	-	3.85	-	3.33	-	3.74	-	3.61	-	3.57	-

[a] Four-point response scale
